# A region growing and local adaptive thresholding-based optic disc detection

**DOI:** 10.1371/journal.pone.0227566

**Published:** 2020-01-30

**Authors:** Tariq M. Khan, Mehwish Mehmood, Syed S. Naqvi, Muhammad Fasih Uddin Butt

**Affiliations:** Department of Electrical and Computer Engineering, COMSATS University, Islamabad, Pakistan; University of California San Diego, UNITED STATES

## Abstract

Automatic optic disc (OD) localization and segmentation is not a simple process as the OD appearance and size may significantly vary from person to person. This paper presents a novel approach for OD localization and segmentation which is fast as well as robust. In the proposed method, the image is first enhanced by de-hazing and then cropped around the OD region. The cropped image is converted to HSV domain and then V channel is used for OD detection. The vessels are extracted from the Green channel in the cropped region by multi-scale line detector and then removed by the Laplace Transform. Local adaptive thresholding and region growing are applied for binarization. Furthermore, two region properties, eccentricity, and area are then used to detect the true OD region. Finally, ellipse fitting is used to fill the region. Several datasets are used for testing the proposed method. Test results show that the accuracy and sensitivity of the proposed method are much higher than the existing state-of-the-art methods.

## 1 Introduction

Recently, some eye diseases such as diabetic retinopathy (DR) require an automatic system to screen a large number of patients at regular intervals. Image processing techniques are found suitable for the analysis of fundus images (i.e. digital eye images). Fundus image analysis is a challenging task as fundus images contain variable colors. Additionally, the morphology of retinal structures and specific features in various patients may lead to incorrect diagnosis. Several research efforts have been made in the past that deal with detecting retinal parts like an optic disc (OD), blood vessels, field of view (FOV) and retinal lesions containing exudates, microaneurysms, and hemorrhages [[1]–[2]].

Retinal vasculature can be damaged due to eye-related diseases such as DR which is a common diabetic disease and a leading cause of blindness. The retina of the eye is badly affected when tiny blood vessels are damaged due to hypertension and high sugar level resulting in vision loss problems. The World Health Organization (WHO) predicts that over the next 25 years the number of people with diabetes will increase from 130 million to 350 million [3]. As stated by the National Eye Institute database of the United States, diabetes is a significant source of blindness among the 20 to 74 years old individuals. Diabetes is a fast-growing disease in developed as well as underdeveloped countries. It has been estimated that 75 percent of people with DR are living in developing countries, due to insufficient treatment and improper healthcare management. It is worth mentioning that the identification of disease pattern in an early stage is imperative to provide better treatment options.

Numerous ophthalmic pathologies like Glaucoma can be detected by the change in OD shape or color [4, 5]. Localization of optic disc is essential for identification and segmentation of the pathological and anatomical structures in retinal images. It helps to prevent false positive detection of exudates incurred by DR since both form a bright region in fundus images [6]. The center of the OD or optic nerve head can be used as a starting point for vessel detection techniques [5].

OD localization is a challenging task due to the variation of OD’s color and shape. Different algorithms are used to localize OD by finding high-intensity value pixels. Abramoff *et. al*. [7] and Patton *et. al*. [8] gave a detailed review of different methods for OD segmentation.

Mathematical morphology and watershed transformation combination were applied for OD detection by Walter *et. al*. [9]. In this scheme, the contrast of hard exudates is decreased and the slow variations of the background are eliminated using a shade correction technique. Neighboring pixels’ local gray-level intensity variance was applied to the shade corrected image for OD locus estimation. OD boundaries are located using watershed transformation. Welfer *et. al*. [10] used adaptive morphological approach for the segmentation of the OD region. Morales *et. al*. [6] used principal component analysis (PCA) for gray conversion for obtaining a gray image. Watershed regions are extracted by applying stochastic watershed on the gray image. Pixels which belong to the OD region are extracted using region discrimination based on the average intensity of the region.

Joshi *et. al*. localized OD using a hybrid approach based on intensity and vessel structure [11]. First, curvature information is used to derive the locations of candidate optic disc that encodes intensity features. Accordingly, the location of a candidate having the maximum value was recognized as an OD. Osareh *et. al*. [12] suggested localization of OD based on the template. Color normalization of retinal images is used in this method followed by template matching. Ying *et. al*. [13] used a two-step strategy for OD localization. Firstly, all the bright regions in a local surrounding were determined to choose the candidates for the OD region. Secondly, a scheme offered by Zhang *et. al*. [14] is used to obtain binary blood vessels skeleton map. In this method, the candidate OD region with the maximum fractal dimension was nominated as OD. The algorithms proposed by [15], [16] and [17] failed to localize the optic disc in case of large bright lesions present in fundus images. Suitable results were obtained from these methods only in typical fundus images having bright and visible OD region. Osareh *et. al*. [12] assumed that the OD is nearly circular and contains bright pixels. This algorithm was unsuccessful where the OD in the retinal image is not the largest and the brightest area.

In this paper, a novel approach has been proposed that is fast and more accurate than the existing state-of-the-art unsupervised OD detection methods. First, the image is enhanced by de-hazing then it is cropped using morphological operations. The V channel of the cropped image is selected and vessels are removed using Laplace transform. The vessels free channel is first thresholded using local adaptive thresholding and then by region growing. Finally, the true OD region is extracted using two region properties i.e area and eccentricity by finding the most eccentric region in the defined range of area from the output of both thresholded images. The main contributions of this paper are:

1. The idea of haze lines is effectively exploited for haze removal and contrast enhancement of OD images, which may include haze or poor contrast due to various leakages occurring from DR.
2. Selection of V channels performed much better than other channels.
3. The Laplace transformation is adapted for effective vessel inpainting and removal.
4. An efficient adaptive local thresholding and region growing-based thresholding method is used for isolating the OD region.

The paper is organised as follows. Section 2 explains the implementation of the proposed method. Section 3 presents the results and experimental outcomes. Section 4 presents the discussion and conclusion.

## 2 Proposed optic disc segmentation algorithm

**Fig 1:**
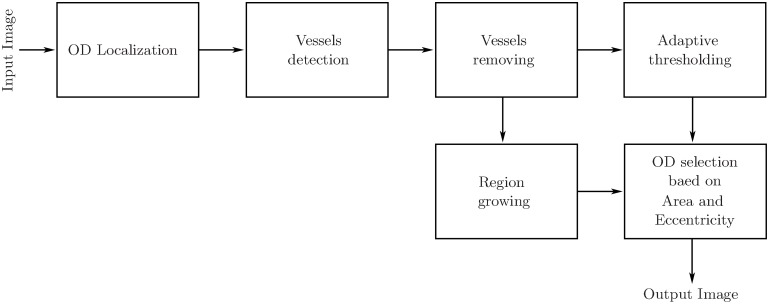
Block diagram of the proposed OD segmentation method.

Detection of OD is a challenging task due to variation in color and shape of an OD. Segmentation of OD is problematic as some disc boundary parts are not properly defined. Therefore, detection of the disc boundary is aimed for correct OD segmentation by identifying the boundary between the nerve head and retina. Fig 1 shows the block diagram of the proposed method.

### 2.1 OD localization

**Fig 2:**
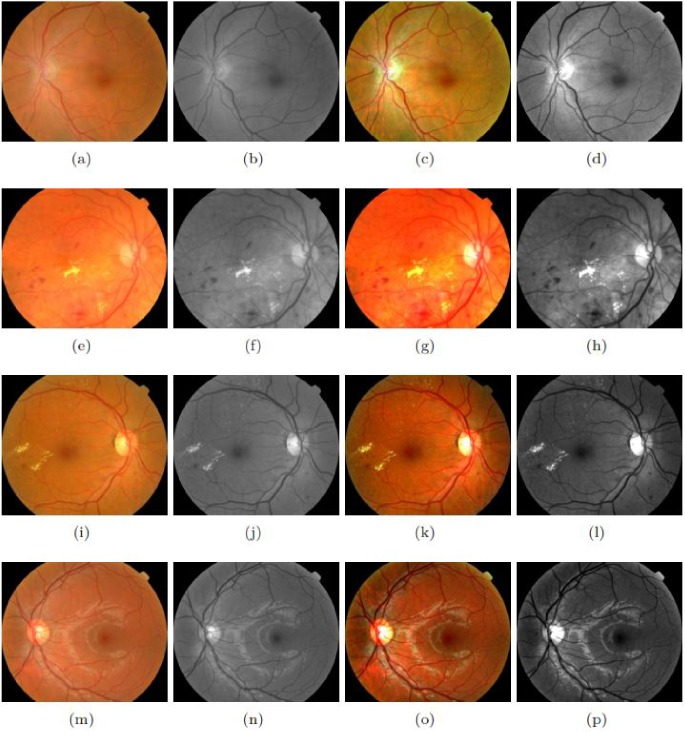
Impact of De-hazing on the retinal image: First column (a, e, i, m) shows four samples of retinal images. In the second column (b, f, j, n) the green channels of first column are shown. In the third column (c, g, k, o) the output of De-hazing is shown. In the fourth column (d, h, l, p) the green channels of third column are shown.

The intensity variations inside the optic and outside the disc cause non-uniform illumination. The region with varied contrast can be modeled as haze with less visibility. For greater clarity of the OD region, a de-hazing algorithm is used for improving the visibility of images captured in outdoor scenarios [18]. This is a non-local method that removes haze from an image. Our objective here is to restore the true color of the OD in the presence of noise, e.g. white lesions, strongly visible choroidal vessels and light artifacts. A hazy image is a complex combination of a true scene, vessels and OD in our case, and global error (non-uniform illumination). This phenomenon fits well into the haze model. It has been observed that a haze-free image in the RGB domain can be represented well with few-hundred distinct colors, that form spherical clusters in RGB space. The presence of haze in images modifies the clusters into a straight-line shape, referred to as haze-lines. Using the haze-lines, a regularized inverse algorithm is presented to make the image haze-free. We adopted this inverse process for its contrast-improvement behavior and efficient computational structure nature that is linear in size to the image. Fig 2 shows the impact of de-hazing on color fundus images.

After de-hazing, the green channel is selected, since vessels are more prominent in this channel [19, 20]. The effect of vessels is first minimized using morphological dilation operation. A line type structure element of length 12 is applied. As the vessels direction varies in a retinal image therefore, the application of angular dilation filters is found to be more effective. A total of 15 dilation filters are applied and their max response is obtained. The aim of removing vessels is to minimize the effect of vessels in the OD region. To get rid of other undesirable features (light artifacts or diseases e.g. exudates, white lesions, etc.), two morphological operations, erosion followed by dilation are used. As the shape of OD is almost like a disc, therefore the structuring element of type disc is applied on the de-hazed image. The size of the disc is set empirically as 15 for erosion and 20 for dilation. This formation is found to be effective for removing undesirable features. After morphological operations, the image is thresholded using Otsu’s [21] thresholding. After thresholding, the centroids and eccentricities of the remaining region are computed. The centroid of the region having minimum eccentric value is picked for cropping. A 300 × 300 sub-image which includes OD is cropped based on the detected point. From this cropped image a circular region of radius (*R* = *R*_*a*_ + 0.3*R*_*a*_) is created, where *R*_*a*_ is the average Radius of OD region of a particular dataset. The resultant image is used for the subsequent operations.

### 2.2 Line detector for vessel extraction

If we divide a retinal image into uniform and non-uniform illumination areas then OD falls in the non-uniform area, so if we can extract only the non-uniform illumination from an image then the process of extracting an OD will be much easier than extracting it from the original image. To do this task effectively, the image is transferred to the HSV domain from the RGB domain. Unlike RGB, HSV separates the image intensity from the color information. Although using both Green and S channels, we can get in the OD region but the perfect reconstruction of the disc in Green channel is hard that may require complex image processing techniques. While in V channel a simple thresholding technique can give a perfect disc. This reduces the computation time. Therefore, for further processing V channel is selected.

In this subsection, the process of extraction of vessels from the Green channel is explained. If we observe the V-channel, we can see some vessels are still present in it. Removing these vessels will further make the process of OD extraction easy. To remove these vessels the best way is to first truly locate these vessels. The best image that possesses most of the vessels is the Green channel [22]. Therefore, for vessels extraction, a multi-scale line detector is applied on the Green channel. The algorithm is proposed by Nguyen et.al. [23] in which the length of the aligned lines is varied to simplify the basic line detector.

### 2.3 Vessels filling by laplace interpolation

Vessels filling is an essential task to correctly estimate the true size of the OD. This filling process of a certain region is often called “inpainting”. A fast and simple method to estimate the missing values of the vessel region is the Laplace transform. It uses a neighbor indexing technique for estimating missing pixels. For estimating missing pixels we can write:

$$\begin{array}{c} {\hat{E}}_{0}=\frac{1}{4}(E_{n}+E_{s}+E_{e}+E_{w}), \end{array}$$ (1)

where ${\hat{E}}_{0}$ denotes the shading estimate of any particular missing pixel. The subscripts *n*, *s*, *e* and *w* denote the four adjacent pixels.

Filling region with Laplace process can effectively be interpreted as a differential mean, as the missing values are modeled as an average of the adjacent pixel values. The same procedure is applied on S-channel for vessels filling.

### 2.4 Adaptive thresholding

Gaussian filter which is also called Gaussian blur or Gaussian smoothing is used to estimate the local background. Selection of *σ* depends on the size of the OD. It should be much smaller than the OD radius that is usually from 30-50 pixels. Therefore, Gaussian function with the value of *σ* = 7 is used. This filter results in blending the prominent structure with the background that produces a blurred image comprising of a slowly-varying illumination pattern. The difference *D*_*I*_ between the image *I* and the mean image *I*_*B*_ is calculated for every pixel:

$$\begin{array}{c} D_{I}(x,y)=I(x,y)-I_{B}(x,y). \end{array}$$ (2)

The resultant is a high-pass filter that passes the structure of interest which has a uniform background. If we threshold this background subtracted image at 0, the resultant image is a sign image.

#### 2.4.1 Morphological operation (erosion and dilation)

The high-pass filtered image posses the OD region and some noise around the OD. This can be easily removed using morphological operations. To do this, two morphological operators, erosion and dilation, are used. As the OD is a circular region, a circular structuring element is used. First, an erosion operation is applied using a circular disc of diameter 13 pixels as the structuring element. This is followed by dilation using a circular disc of diameter 12 pixels. The selection of diameters for erosion and dilation is not as critical as the noise around the OD is consistent.

#### 2.4.2 Region growing

The basic concept of region growing is that the region is iteratively grown by comparing all unallocated neighboring pixels to the region. The difference between a pixel’s intensity value and the regions mean or initial seed value is used as a measure of similarity. The initial seed is assigned by finding the centroid of the localized OD. The pixel with the smallest difference measured this way is allocated to the region. This process stops when the intensity difference between the region and a new pixel becomes larger than the threshold value of 0.05.

### 2.5 OD selection based on area and eccentricity

A unique label is assigned to each connected region of both outputs (region growing adaptive thresholding) so that different objects can be distinguished. After that, the object features are extracted based on the label. Eccentricity *E* of each component can be calculated by:

$$\begin{array}{c} E=\sqrt{\frac{M_{j}^{2}-M_{n}^{2}}{M_{j}^{2}}}. \end{array}$$ (3)

*M*_*j*_ and *M*_*n*_ are major and minor axis lengths. A region in the selected area with smallest *E* values is selected as OD region. The range of area is defined as *A* = *A*_*a*_∓0.35*A*_*a*_, where *A*_*a*_ is the average area of OD region of a particular database.

### 2.6 Ellipse fitting

Once the OD region is extracted, due to noise and the processes of vessel removing, its boundary can be of irregular shape. Using this image for analysis can result in lower values for the measuring parameters. To mitigate these effects and estimate the true OD region, ellipse fitting [24] is computed as the OD boundary. In ellipse fitting, a Least-Squares criterion is used to estimate the best fit to an ellipse from a given set of points. This process of ellipse fitting minimizes the noise effects introduced by vessels especially inside or outside of the OD region.

## 3 Databases

To verify the effectiveness of the proposed method and compare its results with the existing state-of-the-art methods six databases are used namely: DRIONS, MESSIDOR, ONHSD, DIARETDB1, DRISHTI and RIM-ONE.

### 3.1 DRIONS database

DRIONS database comprises of 110 OD segmented digital color fundus images by two different experts. The images were obtained by a color analogical fundus camera and digitized using an HP-Photo Smart-S20 high-resolution scanner. It has 600 × 400 resolution and 8 bits per pixel. This database contains Caucasian patients with an average age of 53 years and a female and male ratio of 53.8% and 46.2%, respectively. 23.1% patients had prolonged modest glaucoma and 76.9% eye hypertension. A portion of 110 images contain visual attributes identified with potential issues that may mutilate the OD contour identification procedure [25]. Table 1 shows Visual characteristics of DRIONS database.

**Table 1:** DRIONS database visual characteristics.

Characteristics	Number of images
Light Artifact	3
Rim Blurred or Missing	5
Moderate Parapapillary Atrophy	16
Concentric Parapapillary Atrophy	20
Strong Pallor Distractor	6

### 3.2 MESSIDOR database

The MESSIDOR database consists of 1200 color images. Three ophthalmologic departments were involved in acquiring these images. A color video 3CCD (Charge Coupled Device) camera is used on a Topcon TRC NW6 non-mydriatic retinograph with 45-degree field of view. The images were captured with three different resolutions at 1140 × 1960, 2240 × 1488 or 2304 × 1536 pixels using 8 bits per color plane. Out of 1200 images, 800 images were acquired with pupil dilation and 400 without dilation [26, 27]. The ground truth of OD boundaries of these 1200 images have been segmented and are currently available online [28].

### 3.3 ONHSD database

The ONHSD database comprises 99 fundus images having 640 × 480 resolution acquired from 50 patients sampled randomly from a DR screening programme. 90 images are designated for assessing segmentation algorithm and 96 images have visible optic nerve head (ONH). These images were taken by a Canon CR6 45MNf fundus camera, having the field angle lens of 45 degrees. There is a significant dissimilarity in the images, with many characteristics that can disturb the algorithm. They are summarized in Table 2. In this database, the center of ONH has been marked up by a doctor, and four doctors marked the ONH edge that intersects with radial bars (at 15-degree angles) radiating from the selected center. The mean of the edges marked by the four specialists has been used to produce the best standard.

**Table 2:** ONHSD database visual characteristics.

Characteristics	Number of images
Light Artifact	7
Rim Blurred or Missing	27
Moderate Parapapillary Atrophy	29
No detectable optic nerve head	4
Severe Cataract	8
Moderate Cararact	2
Exudates or laser scars	7
Easily visible choroidal vessels	20
Severe peripapillary atrophy	6
Concentric Parapapillary Atrophy	23
Strong Pallor Distractor	13

### 3.4 DIARETDB1 database

The DIARETDB1 is a public database which is used to benchmark the diabetic retinopathy detection method. It consists of 89 color fundus images. Out of 89 images, 5 images are normal and 84 contain at least mild non-proliferative signs (Microaneurysms) of the diabetic retinopathy. A 50-degree field-of-view digital fundus camera is used to acquire these images.

### 3.5 DRISHTI database

The DRISHTI dataset has 101 images of which 31 are normal and 70 are abnormal. These images are produced with 30° degree field of view and have a resolution of 2896 × 1944. The OD is correctly marked by four glaucoma experts for each image.

### 3.6 RIM-ONE database

RIM-ONE is a publicly available dataset of retinal images. It consists of 455 high-resolution images of which 255 images are normal and 200 images are abnormal (belong to patients with glaucoma).

## 4 Quantitative performance measures

**Fig 3:**
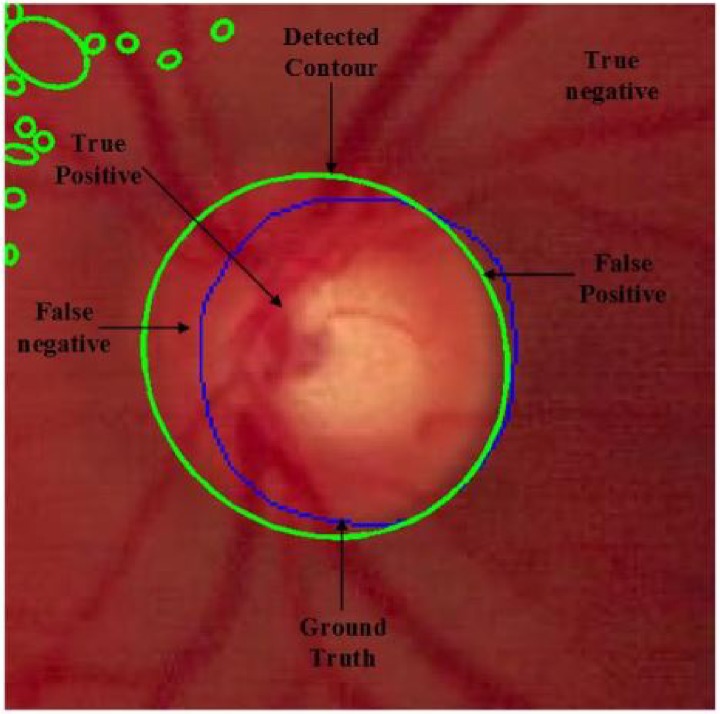
Final extracted image, blue lines represents OD of ground truth and green represents extracted OD of the proposed method.

This paper presents an algorithm for OD localization and OD detection which has been verified using six databases: DIARETDB1, MESSIDOR, DRIONS, DRISHTI, RIM-ONE and ONHSD. To assess the performance of the proposed method different quantitative metrics are used. These quantities are shown in Fig 3.

The process of OD detection and segmentation enables to classify the pixels belonging to the OD region or non-OD region. For pixel classification, there are four possibilities, as illustrated in Table 3: True Negative (TN), True Positive (TP), False Negative (FN) and False Positive (FP). The first two indicate the mutual agreement between predicted values and actual values while the last two indicate a wrong prediction. When the system predicts that the pixel belongs to the OD and is actually an OD pixel then this is the case of TP. When the system predicts that the pixel belongs to the non-OD and is actually a non-OD pixel then this is the case of TN. FP is the case when a pixel actually belongs to the non-OD region while the system predicts the pixel as an OD pixel. Finally, in the FN case the system predicts a pixel as a non-OD pixel when it actually is an OD pixel. Table 4 describes the metrics used to evaluate the quantitative performance of the proposed methodology. In the proposed method, four measures are used namely: Sensitivity (SN), Specificity (SP), Accuracy (Acc) and area overlap (AOL). The overlap metric is defined in Eq 4.

$$\begin{array}{c} AOL=\frac{TP}{\left(FP+TP+FN\right)}. \end{array}$$ (4)

**Table 3:** Confusion matrix.

	OD Available	OD Not Available
Classify correctly	TP	FP
Classify wrongly	FN	TN

**Table 4:** Performance metric for OD segmentation.

Measure	Description
SN	TP/(TP + FN)
SP	TN/(TN + FP)
Acc	(TP + TN)/(TP + FP+ TN + FN)
AOL	TP/(TP + FP + FN)

## 5 Experimental results

The pixel-wise quantitative performance metrics, as described in Table 4, are calculated for OD segmentation. The methodology is quantitatively evaluated by using these performance metrics.

### 5.1 Localization result

Most of the existing methods, including [25, 29] and [5] used localization. In this paper, we have proposed a fast and robust technique that gives promising results. Table 5 compares the result of the proposed method with [5]. It can be observed that the accuracy of the proposed method on MESSIER and DRIONS databases which is 99% and 100% respectively, is better than [5].

**Table 5:** Localization percentage of OD using green channel of fundus image.

Localization	MESSIDOR	DRIONS DB	ONHSD
[5]	97%	98%	99%
Proposed	99%	100%	99%

### 5.2 Comparison of performance measures with other algorithms

The comparison of the proposed method has been made with other available methods on the basis of average sensitivity, specificity, accuracy, and AOL. Six retinal images datasets namely DROINS, DIARETDB1, MESSIDOR, DRISHTI, RIM-ONE and ONHSD are used.

**Fig 4:**
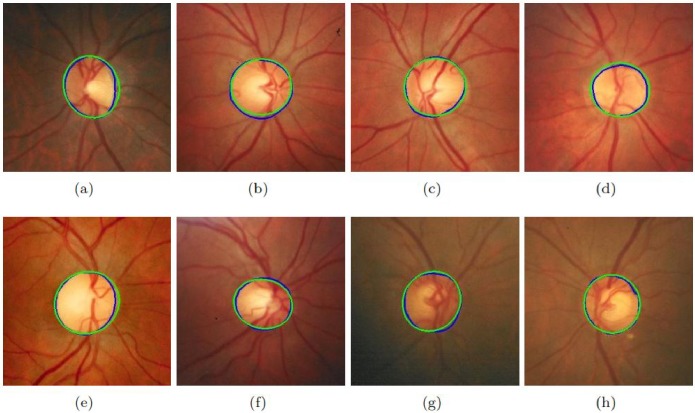
Results of the proposed method on MESSIDOR dataset. (a)-(h) shows the performance of the proposed method on eight selected images. Green color shows the proposed method’s detected OD while blue color is of ground truth.

**Fig 5:**
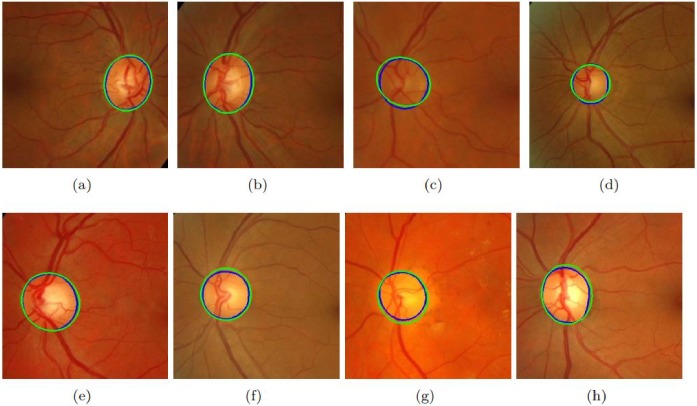
Results of the proposed method on DRION dataset. (a)-(h) shows the performance of the proposed method on eight selected images. Green color shows the proposed method’s detected OD while blue color is of ground truth.

**Fig 6:**
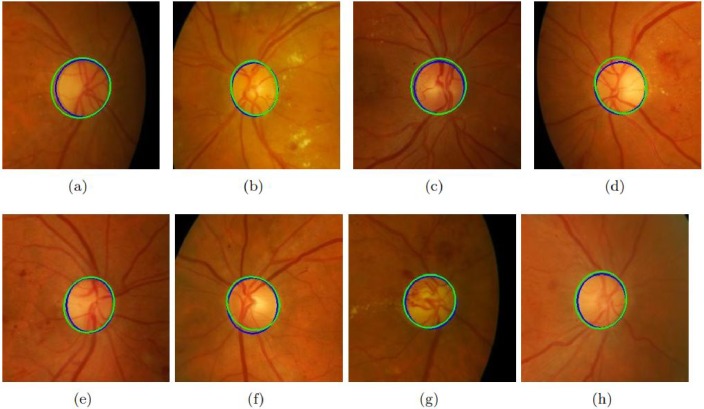
Results of the proposed method on DIARETDB1 dataset. (a)-(h) shows the performance of the proposed method on eight selected images. Green color shows the proposed method’s detected OD while blue color is of ground truth.

**Fig 7:**
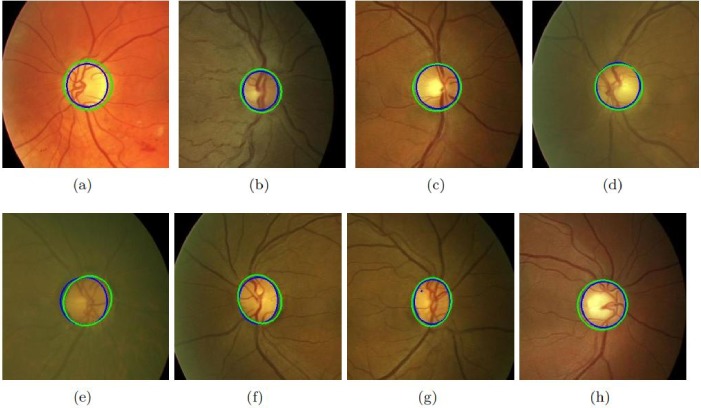
Results of the proposed method on ONHSD dataset. Fig (a)-(h) show the performance of the proposed method on eight selected images. Green color shows the proposed method’s detected OD while blue color is of ground truth.

Different approaches have been selected for comparison including both supervised and unsupervised. The results of these datasets are shown in Table 6. It must be noted that the proposed method’s performance in terms of accuracy is much better than all existing state-of-the-art methods. Fig 4 shows the results of the proposed method on sample images of MESSIDOR database. Fig 5 shows the results of the proposed method on sample images of DRIONS-DB database. The sensitivity of the proposed method is higher than all existing state-of-the-art methods on all datasets. If we compare the computational time of OD segmentation of the proposed method with other state-of-the-art than the proposed method is much faster than all other existing methods. Fig 6 shows the results of the proposed method on sample images of DIARETDB1 database. Fig 7 shows the results of the proposed method on sample images of ONHSD database. The AOL of the proposed method is comparable to the state-of-art methods on all given datasets.

**Table 6:** OD segmentation performance measures comparison with other methods.

Performance measures	Sensitivity	Specificity	Accuracy	AOL	Average time per image (in sec)
**Methods**					
**DIARETDB1**					
[30]	0.8808	0.9878		0.334	120.5
[31]	0.8498	0.9964		0.34	59.72
[32]	0.7347	0.9944		0.546	
[33]	0.6341	0.9983		0.391	
[34]	0.851	0.9984	0.9772	0.851	40
**Proposed method**	**0.9337**	**0.9965**	**0.9950**	**0.8359**	**1**
**MESSIDOR**					
[6]	0.93		0.9949	0.8228	
[25]	0.9212		0.977	0.8636	
[34]	0.8954	0.995	0.9989	0.8793	71.3
**Proposed method**	**0.9291**	**0.9953**	**0.9921**	**0.8357**	**1**
**DRIONS-DB**					
[25]	0.8957		0.976	0.8473	
[34]	0.8508	0.9995	0.9989	0.851	43.2
**Proposed method**	**0.9441**	**0.9956**	**0.9931**	**0.8589**	**1**
**Drishti-DB**					
**Proposed method**	**0.9649**	**0.9975**	**0.9960**	**0.9179**	**1**
**RIM-ONE-DB**					
**Proposed method**	**0.9611**	**0.9932**	**0.9909**	**0.8568**	**1**
**ONHSD**					
[25]	0.9077		0.9895	0.8346	
[6]	0.931		0.9941	0.8045	
**Proposed method**	**0.9341**	**0.991**	**0.9899**	**0.8112**	**1**

### 5.3 Limitations

**Fig 8:**
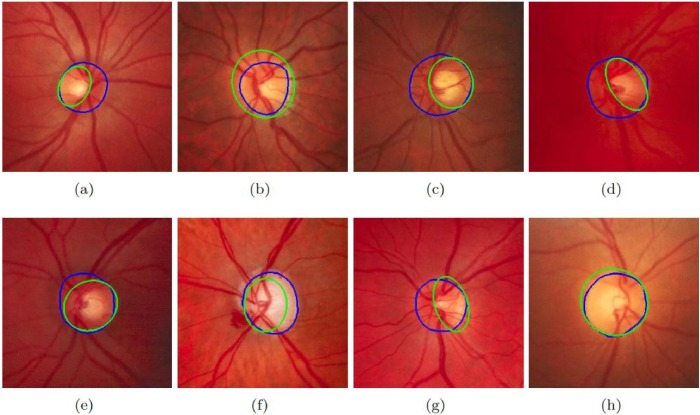
Fig (a)-(h) show the performance of the proposed method on eight difficult selected images. Green color shows the proposed method’s detected OD while blue color is of ground truth.

In the proposed OD segmentation method, two morphological operations namely erosion and dilation have been used to remove noise. The diameter selection of disc does have same effect on the OD segmentation either when the outer region of OD is affected by disease or the inner region has varied contrast. Although the selection V channels overcomes these effects, but due to the uncalibrated nature of the original color input image, in some cases, we found that the proposed method either segments partial OD or segments some outer regions with OD, as shown in Fig 8.

## 6 Discussion and conclusion

In this paper, a novel method for OD localization and segmentation is proposed. The method presented a robust approach for OD localization. For localization of OD, the image is first de-hazed and then using morphological operation a most eccentric region is found. For OD segmentation, a hybrid approach is used in which both S and V-channel are processed in parallel. Finally, the OD is segmented using eccentricity and area properties. The method is evaluated on four retinal image datasets. Experimental evaluation shows that this method is computationally fast, robust to the variations in image contrast and illumination, works well in pathological retinal images and is comparable with state of the art methodologies in terms of quantitative performance metrics. The proposed method achieved about 99% accuracy on four datasets.

The results of OD segmentation illustrate the ability of the proposed algorithm for localization when the images are affected by light artifacts such as when the OD boundary is impacted with lesions of retinopathy that may lead towards the false positive prediction. The robustness of the proposed method is also evaluated using these light artifact images. The visual and quantitative analysis shows the success of our proposed method. Furthermore, the method successfully segments the OD with these pathological structures.

In the future, we aim to develop an automated method for glaucoma screening. Even though related published works can be used, our work shows low computational time, high accuracy, tolerance for different varieties of low contrast images and can be easily integrated with glaucoma and other radiography lesion systems.
